# Association between rapid serum sodium correction and rhabdomyolysis in water intoxication: a retrospective cohort study

**DOI:** 10.1186/s40560-017-0233-0

**Published:** 2017-06-19

**Authors:** Masahiro Kashiura, Kazuhiro Sugiyama, Yuichi Hamabe

**Affiliations:** Emergency and Critical Care Center, Tokyo Metropolitan Bokutoh Hospital, 4-23-15 Kotobashi, Sumida-ku, Tokyo, 130-8575 Japan

**Keywords:** Osmotic demyelination syndrome, Hyponatremia, Myoglobinuria, Polydipsia, Seizures

## Abstract

**Background:**

Patients with water intoxication may develop rhabdomyolysis. Existing studies suggest a relationship between the serum sodium correction rate and rhabdomyolysis. The aim of the present study was to determine the association between the sodium correction rate and rhabdomyolysis in patients with water intoxication.

**Methods:**

Medical records from all cases of water intoxication presenting to the emergency department and admitted to a single tertiary emergency hospital between September 2012 and August 2016 were examined retrospectively. Serum sodium correction rate was defined as the difference in serum sodium levels at admission and approximately 24 h after admission, divided by time. The primary outcome was rhabdomyolysis, defined as peak creatine kinase level ≥ 1500 IU/L. Logistic regression analysis was used to calculate the adjusted odds ratio of the serum sodium correction rate controlling for age, sex, convulsion, lying down for >8 h before admission to the emergency department, and serum sodium level on admission.

**Results:**

A total of 56 cases of water intoxication were included in the study. The median serum sodium correction rate was 1.02 mEq/L/h, and 32 patients (62.5%) had rhabdomyolysis. Logistic regression analysis showed that serum sodium correction rate was an independent risk factor of rhabdomyolysis (adjusted odds ratio, 1.53 per 0.1 mEq/L/h; 95% confidence interval, 1.18–1.97).

**Conclusions:**

Rapid correction of serum sodium was associated with rhabdomyolysis in patients with water intoxication. Therefore, strict control of serum sodium levels might be needed in such patients.

## Background

Water intoxication is a common comorbidity associated with mental disorders such as schizophrenia and dementia [1]. Hyponatremia induced by water intoxication can cause headache, seizures, and disturbance of consciousness [2]. The principal treatment of hyponatremia in cases of water intoxication is fluid restriction. However, rhabdomyolysis may complicate the situation during treatment of hyponatremia induced by water intoxication [3–6]. Two observational studies have suggested that the sodium correction rate is associated with rhabdomyolysis [7, 8]. However, these were exploratory studies and had insufficient sample sizes to adjust for possible confounders such as age, sex, and presence of seizures. As such, the association between the serum sodium correction rate and rhabdomyolysis in cases of water intoxication remains unclear [7, 8]. Therefore, this study aimed to determine this association while controlling for possible confounders.

## Methods

### Study design and setting

This retrospective cohort study was conducted in a single tertiary emergency medical center covering the local population of approximately 1,800,000 in the east Tokyo metropolitan area of Japan.

### Participants

Data from patients with hyponatremia induced by water intoxication who presented to the emergency department and who were admitted to our hospital between September 2012 and August 2016 were extracted from medical records retrospectively. The diagnosis of water intoxication consisted of hyponatremia (≤125 mEq/L), polydipsia (> 6 L/day), and exception of other disorders such as syndrome of inappropriate secretion of antidiuretic hormone. Patients with low creatine kinase (CK) levels (<1500 IU/L) at admission were included. Exclusion criteria were as follows: (i) non-admittance; (ii) discharge or transfer to another hospital within 24 h of admission; and (iii) presence of chronic heart failure, liver cirrhosis, or chronic kidney disease. The treatment of water intoxication was water restriction in most cases. Infusion rate and contents of fluid administration were modified according to serum electrolyte level and urine output at the discretion of the physician in charge.

### Exposure and comparison group definitions

Serum sodium level was measured every 4 h, at least during the first 24 h after admission. The serum sodium correction rate was defined as the difference in serum sodium level at admission and approximately 24 h after admission, divided by time (e.g., in case of 110 mEq/L at admission and 130 mEq/L at 20 h after admission, the serum sodium correction rate is 1.0 mEq/L/h). As a sensitivity analysis, the serum sodium correction rates during the first 12 and 48 h were calculated similarly.

### Data collection and definitions

The following data were collected from medical records: age, sex, convulsions, medical history of mental disease, lying down for >8 h before admission, transition of laboratory test values (serum electrolytes, CK, and creatinine), hospital stay, complications during the clinical course such as acute kidney injury and osmotic demyelination syndrome, and death. Immobilization for a long duration is a risk factor for non-traumatic crash syndrome. Therefore, we considered 8 h as a clinically important risk of crash syndrome and lying down for >8 h before admission was measured as a potential confounder.

### Outcome measurements

The primary outcome was rhabdomyolysis. There is no standard definition of rhabdomyolysis [9]; therefore, rhabdomyolysis was defined as peak CK level ≥ 1500 IU/L (ten times the upper limit of the normal range at our institution) with reference to muscular injury due to statin use, as per the guidelines of the American College of Cardiology/American Heart Association/National Heart, Lung and Blood Institute [10]. Secondary outcomes included mortality, duration of hospital stay, and presence of complications such as acute kidney injury and osmotic demyelination syndrome. Acute kidney injury was staged according to the Kidney Disease Improving Global Outcomes clinical practice guidelines [11].

### Statistical analysis

Descriptive statistics were calculated for all variables of interest. Continuous variables are reported as medians and interquartile ranges, and categorical variables are presented using counts and percentages. Univariate analysis was performed using Fisher’s exact test or chi-square test, as appropriate, for binary variables and Mann-Whitney *U* test for continuous and ordinal variables for comparison of the two groups. Correlation between serum sodium correction rate and peak CK level was assessed using Spearman’s rank correlation coefficient and simple linear regression analysis. Multivariate logistic regression analysis for rhabdomyolysis occurrence was conducted with adjustment for predetermined potential confounders (age, sex, convulsions, lying for >8 h before admission to the emergency department, and serum sodium level on admission). For the sensitivity analysis, multivariate logistic regression analysis was performed similarly, using the serum sodium correction rates at 12 and 48 h after admission. In addition, the receiver operating characteristic (ROC) curve was described, and the area under the curve (AUC) was calculated to estimate the predictive values of the serum sodium correction rate for rhabdomyolysis. We determined the cutoff point to maximize the predictive power of Youden’s index. All reported *p* values were two-tailed, and *p* values <0.05 were considered statistically significant. Statistical analysis was performed using IBM SPSS for Mac Version 22.0 (IBM Corp., Armonk, NY).

## Results

During the study period, 57 cases of water intoxication met the inclusion criteria. Of these, one patient was excluded due to ongoing maintenance hemodialysis. Thus, the final study sample comprised 56 cases.

Participant characteristics and outcomes are shown in Table 1. The median serum sodium correction rate during the first 24 h was 1.02 mEq/L/h. Among the 56 patients, 35 patients (62.5%) had rhabdomyolysis. There were no cases of osmotic demyelination syndrome or death. The median values of the serum sodium correction rate in the cases with rhabdomyolysis in 12 and 24 h were higher than those in the cases without rhabdomyolysis (1.22 vs. 0.71 mEq/L/h, 1.11 vs. 0.60 mEq/L/h, *p* < 0.001, respectively). In addition, the duration of hospital stay was significantly longer and the incidence of acute kidney injury was higher in the cases with rhabdomyolysis (*p* < 0.001 and = 0.012, respectively).

**Table 1:** Baseline characteristics and outcomes of the study sample

	Overall	Rhabdomyolysis (+)	Rhabdomyolysis (−)	*p* value
	(*n* = 56)	(*n* = 35)	(*n* = 21)	
Age, years	53.0 [42.0, 62.0]	47.0 [41.0, 59.5]	58.0 [47.0, 63.0]	0.056
Male sex	26 (46.4)	21 (60.0)	5 (23.8)	0.013
Underlying mental disorder	51 (91.1)	31 (88.6)	20 (95.2)	0.64
Convulsion	34 (60.7)	25 (71.4)	9 (42.9)	0.049
Lying down for >8 h before admission	43 (76.8)	30 (85.7)	13 (61.9)	0.054
Serum CK level on admission, IU/L	450 [194, 775]	661 [308, 944]	215 [78, 404]	<0.001
Serum creatinine level on admission, mg/dL	0.60 [0.50, 0.70]	0.60 [0.50, 0.70]	0.50 [0.50, 0.70]	0.75
Serum sodium level on admission, mEq/L	110.5 [108.0, 113.3]	110.0 [108.0, 112.5]	111.0 [109.0, 116.0]	0.13
Serum sodium correction rate during first 12 h, mEq/L/h	1.15 [0.74, 1.31]	1.22 [1.11, 1.39]	0.71 [0.61, 0.85]	<0.001
Serum sodium correction rate during first 24 h, mEq/L/h	1.02 [0.63, 1.20]	1.11 [0.97, 1.27]	0.60 [0.50, 0.74]	<0.001
Serum sodium correction rate during first 48 h, mEq/L/h	0.58 [0.54, 0.61]	0.58 [0.56, 0.62]	0.57 [0.51, 0.61]	0.19
Muscle pain	4 (7.1)	4 (11.4)	0 (0.0)	0.29
Peak serum CK, IU/L	5249 [822, 21,003]	10,323 [5775, 35,695]	540 [236, 873]	<0.001
Peak serum creatinine, mg/dL	0.70 [0.60, 0.80]	0.70 [0.60, 0.80]	0.60 [0.50, 0.70]	0.14
Hospital stay, days	9.0 [6.0, 11.0]	11.0 [8.5, 12.5]	5.0 [4.0, 8.0]	<0.001
Acute kidney injury				0.012
Stage 1	8 (14.3)	8 (22.9)	0 (0.0)	
Stage 2	1 (1.8)	1 (2.9)	0 (0.0)	
Stage 3	0 (0.0)	0 (0.0)	0 (0.0)	
Osmotic demyelination syndrome	0 (0.0)	0 (0.0)	0 (0.0)	NA
Death	0 (0.0)	0 (0.0)	0 (0.0)	NA

Continuous variables are presented as median [interquartile range]. Categorical variables are presented as counts (percentage)

*CK* creatine kinase, *NA* not applicable

**Fig. 1:**
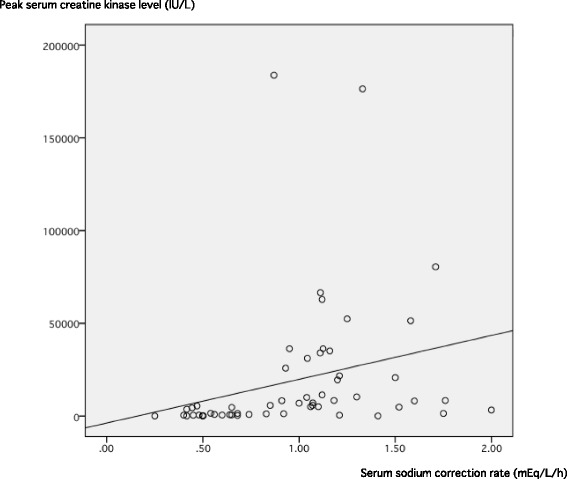
Scatter diagram and regression line between serum sodium correction rate during the first 24 h and peak serum creatine kinase level

Spearman correlation analysis showed a positive correlation between the serum sodium correction rate and peak CK level (*r* = 0.586, *p* < 0.001), and the regression coefficient per 0.1 mEq/L/h was 2361 (*p* = 0.047) (Fig. 1).

Logistic regression analysis for rhabdomyolysis showed the serum sodium correction rate during the first 24 h to be an independent risk factor after adjusting for potential confounders (adjusted odds ratio, 1.53 per 0.1 mEq/L/h, 95% confidence interval, 1.18–1.97) (Table 2). Sensitivity analysis showed that the serum sodium correction rate during the first 12 h was an independent risk factor for rhabdomyolysis, but the coefficient for serum correction rate during the first 48 h was not statistically significant (Table 3).

**Table 2:** Logistic regression analysis of rhabdomyolysis in water intoxication cases

Variable	Odds ratio (95% CI)	*p* value
Age, per 1 year	1.05 (0.97–1.14)	0.23
Lying down for >8 h before admission	20.5 (1.77–237)	0.016
Male sex	19.2 (2.28–161)	<0.001
Convulsion	1.95 (0.31–12.2)	0.47
Serum sodium level on admission, per 1 mEq/L	0.88 (0.75–1.02)	0.092
Serum sodium correction rate during first 24 h, per 0.1 mEq/L/h	1.53 (1.18–1.97)	0.001

*CI* confidence interval

**Table 3:** Sensitivity analysis of logistic regression analysis of rhabdomyolysis using the change in serum sodium level during the first 12 and 48 h

Variable	Odds ratio (95% CI)	*p* value
(A) 12 h		
Age, per 1 year	1.06 (0.97–1.16)	0.22
Lying down for >8 h before admission	15.8 (1.37–181)	0.027
Male sex	20.4 (2.03–205)	0.010
Convulsion	2.32 (0.33–16.4)	0.40
Serum sodium level on admission, per 1 mEq/L	0.86 (0.72–1.02)	0.079
Serum sodium correction rate during first 12 h, per 0.1 mEq/L/h	1.76 (1.28–2.43)	<0.001
(B) 48 h		
Age, per 1 year	1.02 (0.96–1.09)	0.57
Lying down for >8 h before admission	8.63 (1.42–52.4)	0.019
Male sex	9.53 (1.54–59.1)	0.016
Convulsion	3.46 (0.80–15.1)	0.098
Serum sodium level on admission, per 1 mEq/L	0.91 (0.78–1.06)	0.23
Serum sodium correction rate during first 48 h, per 0.1 mEq/L/h	1.32 (0.29–6.11)	0.72

*CI* confidence interval

**Fig. 2:**
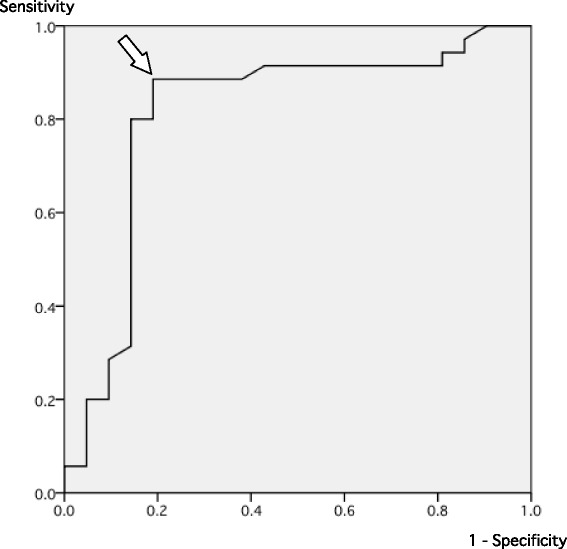
Receiver operating characteristic curve of serum sodium correction rate for rhabdomyolysis. *White arrow* indicates sensitivity and specificity for cutoff value of 0.840 mEq/L/h

The AUC for the ROC curve of the serum sodium correction rate for rhabdomyolysis was 0.812 (Fig. 2). At a cutoff value of 0.840 mEq/L/h, the sensitivity and specificity were 0.886 and 0.810 with the maximum Youden’s index, respectively.

## Discussion

In a retrospective cohort study at a single tertiary emergency medical center in Japan, rapid correction of serum sodium was associated with rhabdomyolysis in patients with water intoxication after adjusting for potential confounding variables. Among the secondary outcomes, hospital stay was longer in the rapid serum sodium correction group, but there were no significant differences in complications or mortality between the two groups.

Our findings are consistent with two previous observational studies suggesting rapid serum sodium correction is associated with rhabdomyolysis [7, 8]. In addition, although rhabdomyolysis is a rare complication of hyponatremia according to previous case reports, rhabdomyolysis would be an under-recognized complication based on our results [5, 12]. Previous studies have suggested that the incidence of rhabdomyolysis in water intoxication patients is approximately 30 to 55% [7, 8].

Two hypotheses regarding the cause of rhabdomyolysis have been described thus far. First, hyponatremia and hyposmolarity may lead to depletion of potassium levels in the muscular system, which in turn may cause rhabdomyolysis [13]. Second, rapid correction of serum sodium levels may lead to failure in regulation of muscular cell volume. As a result, membrane fragility and enzyme leakage may be induced, leading to rhabdomyolysis [7, 14, 15]. In the present study, serum sodium levels at admission did not differ significantly between the rapid and slow serum sodium correction groups. Our results thus support the second hypothesis. Furthermore, in the patients with rhabdomyolysis, the hospital stay was longer and the incidence of acute kidney injury was higher. This result is similar to that of previous studies [7, 8]. Therefore, preventing CK increase by controlling sodium correction rate may lead better outcome of water intoxication patients.

Our analyses showed that both 12- and 24-h serum sodium correction rates were independently associated with rhabdomyolysis. However, analysis of the 48-h serum sodium correction rate did not show any significant difference between the two groups. This may suggest that an early change in the serum sodium level plays a more important role in rhabdomyolysis than does a later change [7]. Therefore, serum sodium level should be strictly controlled in the early phase of hyponatremia treatment. In addition, the ROC in the present study showed that the serum sodium correction rate should be maintained <0.80 mEq/L/h. In general, to prevent osmotic demyelination syndrome, the serum sodium correction should be within 10 to 12 mEq/L in the first 24 h [7]. Therefore, based on the present results, the serum sodium correction rate should be <0.50 mEq/L/h despite no occurrence of osmotic demyelination syndrome. More than half of the subjects failed to achieve such recommendation in the present study. The serum sodium level tends to rise spontaneously in water intoxication unlike syndrome of inappropriate secretion of antidiuretic hormone. Therefore, strict serum electrolyte monitoring should be required, especially in the early treatment phase.

Several limitations of our study must be acknowledged. First, it is a single-center, retrospective observational study. Although this kind of study could not be performed as an interventional study, similar multicenter and prospective studies should be conducted in the future. Second, CK levels at admission were higher in the cases with rhabdomyolysis. Therefore, serum CK level at admission per se might influence the results of the present study. For example, the odds ratio is high for lying down for >8 h before admission, which could lead to non-traumatic crush injury. Therefore, rhabdomyolysis may have occurred before admission. However, the serum sodium correction rate is also an independent risk factor for rhabdomyolysis. Therefore, treatment after admission is also important to prevent rhabdomyolysis. Third, the type of treatment administered, such as fluid intake and sodium administration, was not considered, although almost all patients were treated by water restriction. Finally, it remains unclear whether rapid correction of hyponatremia with or without water intoxication is at a risk of rhabdomyolysis because only cases with hyponatremia due to water intoxication were included in the present study. Despite these limitations, our study has several advantages over previous exploratory studies, including a larger sample size and adjustment for important potential confounders [7, 8].

## Conclusions

In conclusion, rapid correction of serum sodium is associated with rhabdomyolysis in patients with water intoxication. Therefore, strict control of serum sodium levels might be required in such cases.

## Abbreviations

AUC: Area under the curve
CK: Creatine kinase
ROC: Receiver operating characteristic
